# Supplementary material for: The Small RNA Universe of Capitella teleta
Source: Front Mol Biosci. 2022 Feb 25;9:802814. doi: 10.3389/fmolb.2022.802814 (PMC8915122; doi:10.3389/fmolb.2022.802814)
Supplement: Supplementary file 1 [file DataSheet1.ZIP › Supplement/known/CAPTEscaffold_2_206.pdf]

Provisional ID	:	CAPTEscaffold_2_206
Score total	:	46355.1
Score for star read(s)	:	3.9
Score for read counts	:	46346.3
Score for mfe	:	0.3
Score for randfold	:	1.6
Score for cons. seed	:	3
Total read count	:	90918
Mature read count	:	90862
Loop read count	:	0
Star read count	:	56

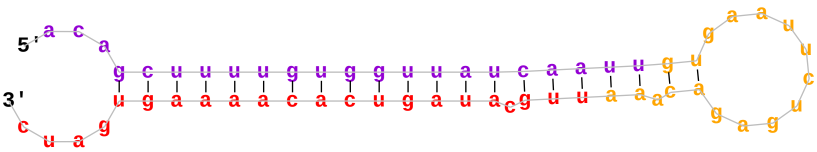

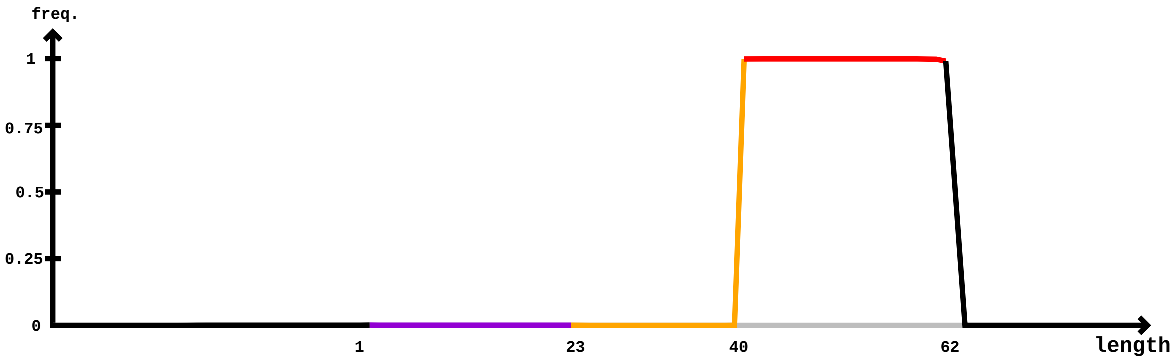

Star Mature

```
5'- uuacauaaucagccuucuuacucggaauacgacagcuuuugugguuaucaauugugaauucugagacaaauugcauagucacaaaagugaucguaaucucaggcaucaccac   -3'   obs
    uuacauaaucagccuucuuacucggaauacgacagcuuuugugguuaucaauugugaauucugagacaaauugcauagucacaaaagugaucguaaucucaggcaucaccac         exp
    uuacauaaucagccuucuuacucggaauacgacagcuuuugugguuaucaauugugaauucugagacaaauugcauagucacaaaagugaucguaaucucaggcaucaccac         known
    ...........((((........(((.(((((..((((((((((((((((((((..........)).))))).))))))))))))))..))))).))).))))........   reads   mm   sample

    ............ccuucuuacucggaauacga................................................................................   8     0    seq
    .............cuucuuacucggaauacga................................................................................   30    0    seq
    .............cuucuuacucggaauacgG................................................................................   1     1    seq
    ..............uucuuacucggaauacga................................................................................   6     0    seq
    ...............................acagcuuuugugguuaucaa.............................................................   1     0    seq
    ...............................acagcuuuugugguuaucaau............................................................   1     0    seq
    ...............................acagcuuuugugguuaucaauu...........................................................   40    0    seq
    ................................cagcuuuugugguuaucaauu...........................................................   14    0    seq
    .....................................................................auugcauagucacaaaagugau.....................   1     0    seq
    .....................................................................auugcauagucacaaaagugauc....................   31    0    seq
    .....................................................................Uuugcauagucacaaaagugauc....................   1     1    seq
    .....................................................................aAugcauagucacaaaagugauc....................   1     1    seq
    .....................................................................Cuugcauagucacaaaagugauc....................   3     1    seq
    ......................................................................uugcauagucacaaaagug.......................   33    0    seq
    ......................................................................uugcauagucacaaaaguA.......................   8     1    seq
    ......................................................................uugcauagucacaaaaguga......................   48    0    seq
    ......................................................................uugcauagAcacaaaaguga......................   1     1    seq
    ......................................................................uugcauagucacaaaagugG......................   2     1    seq
    ......................................................................uugcauaguAacaaaagugau.....................   1     1    seq
    ......................................................................Gugcauagucacaaaagugau.....................   1     1    seq
    ......................................................................uugcauagucacaaaagAgau.....................   1     1    seq
    ......................................................................uAgcauagucacaaaagugau.....................   1     1    seq
    ......................................................................uugcauagucacaaaagugau.....................   642   0    seq
    ......................................................................uugcauagucacaaGagugau.....................   1     1    seq
    ......................................................................uugcauagucacaUaagugau.....................   1     1    seq
    ......................................................................uCgcauagucacaaaagugau.....................   1     1    seq
    ......................................................................Augcauagucacaaaagugau.....................   5     1    seq
    ......................................................................uugcauGgucacaaaagugauc....................   37    1    seq
    ......................................................................uugcauagucacaCaagugauc....................   1     1    seq
    ......................................................................uugGauagucacaaaagugauc....................   5     1    seq
    ......................................................................uugcauagucacaaaagugaGc....................   10    1    seq
```

```
                              Star                                    Mature
uuacauaaucagccuucuuacucggaauacgacagcuuuugugguuaucaauugugaauucugagacaaauugcauagucacaaaagugaucguaaucucaggcaucaccac
......................................................................uugcauagucacaaaaguUauc....................	7	1	seq
......................................................................uugcauagucaGaaaagugauc....................	2	1	seq
......................................................................uugcauaAucacaaaagugauc....................	9	1	seq
......................................................................uugcauagucGcaaaagugauc....................	21	1	seq
......................................................................uugcauagucacaGaagugauc....................	7	1	seq
......................................................................uugcauagucacaaaagGgauc....................	5	1	seq
......................................................................uugcauagucacaaaaAugauc....................	7	1	seq
......................................................................uugcauagucaAaaaagugauc....................	19	1	seq
......................................................................uugcauagucacaaaGgugauc....................	16	1	seq
......................................................................uugcauagucacaaaaCugauc....................	4	1	seq
......................................................................uugcauaguUacaaaagugauc....................	34	1	seq
......................................................................uGgcauagucacaaaagugauc....................	2	1	seq
......................................................................uugcauUgucacaaaagugauc....................	4	1	seq
......................................................................uugcauagGcacaaaagugauc....................	3	1	seq
......................................................................uugcauagucacaaaagugauG....................	27	1	seq
......................................................................uugcauagucacaaaaguCauc....................	6	1	seq
......................................................................uugcauagAcacaaaagugauc....................	30	1	seq
......................................................................uuAcauagucacaaaagugauc....................	12	1	seq
......................................................................uugcauagucacaaaagCgauc....................	16	1	seq
......................................................................uugcGuagucacaaaagugauc....................	18	1	seq
......................................................................uugcauagucacaaGagugauc....................	7	1	seq
......................................................................uugAauagucacaaaagugauc....................	18	1	seq
......................................................................uugcauaguAacaaaagugauc....................	27	1	seq
......................................................................uugcauagucacaaaaguAauc....................	55	1	seq
......................................................................uugcauagucacaaCagugauc....................	1	1	seq
......................................................................uugcauagucacaaaagugauA....................	73	1	seq
......................................................................Cugcauagucacaaaagugauc....................	15	1	seq
......................................................................uugcauaCucacaaaagugauc....................	8	1	seq
......................................................................uugcauagucNcaaaagugauc....................	1	1	seq
......................................................................Nugcauagucacaaaagugauc....................	22	1	seq
......................................................................uugcauagucacGaaagugauc....................	24	1	seq
......................................................................uuCcauagucacaaaagugauc....................	5	1	seq
......................................................................Augcauagucacaaaagugauc....................	368	1	seq
......................................................................uugUauagucacaaaagugauc....................	7	1	seq
......................................................................uugcauagucacaaaagAgauc....................	50	1	seq
......................................................................uugcauagucacCaaagugauc....................	1	1	seq
......................................................................uugcaCagucacaaaagugauc....................	15	1	seq
......................................................................uugcauaguGacaaaagugauc....................	3	1	seq
......................................................................uugcaGagucacaaaagugauc....................	4	1	seq
......................................................................uugcauagCcacaaaagugauc....................	9	1	seq
......................................................................uugcauagucacaaaagugauU....................	146	1	seq
......................................................................uCgcauagucacaaaagugauc....................	20	1	seq
......................................................................uugcauagucacaaaUgugauc....................	4	1	seq
......................................................................uugcauaUucacaaaagugauc....................	4	1	seq
......................................................................uugcauagucacaaaagugaAc....................	31	1	seq
......................................................................uugcauagucacaaUagugauc....................	7	1	seq
......................................................................Gugcauagucacaaaagugauc....................	65	1	seq
......................................................................uugcaAagucacaaaagugauc....................	49	1	seq
......................................................................uugcauagucacaUaagugauc....................	8	1	seq
......................................................................uugcauagucCcaaaagugauc....................	1	1	seq
......................................................................uugcauagucacaaaagugaCc....................	7	1	seq
......................................................................uugcUuagucacaaaagugauc....................	13	1	seq
......................................................................uNgcauagucacaaaagugauc....................	4	1	seq
......................................................................uugcauagucacUaaagugauc....................	8	1	seq
......................................................................uugcauagucacaaaagugGuc....................	10	1	seq
......................................................................uugcauagucacaaaagugauc....................	88272	0	seq
......................................................................uugcauagucacaaaagugUuc....................	9	1	seq
......................................................................uuUcauagucacaaaagugauc....................	18	1	seq
......................................................................uAgcauagucacaaaagugauc....................	90	1	seq
......................................................................uugcauagucUcaaaagugauc....................	13	1	seq
......................................................................uugcauagucaUaaaagugauc....................	18	1	seq
......................................................................uugcauagucacaaaaUugauc....................	5	1	seq
......................................................................uugcauagucacaaaagugaucC...................	1	1	seq
......................................................................uugcauagucacaaaaguAaucg...................	1	1	seq
......................................................................uugcauagucacaaaagugaucg...................	157	0	seq
......................................................................uugcauagucacaaaagugaucA...................	6	1	seq
......................................................................uugcauagucacaaaagugaucU...................	74	1	seq
......................................................................uugcauagucacaaaagugaucgu..................	3	0	seq
......................................................................uugcauagucacaaaagugaucguU.................	1	1	seq
.......................................................................ugcauagucacaaaagugauc....................	7	0	seq
```

Star **Mature**

```
uuacauaaucagccuucuuacucggaauacgacagcuuuugugguuaucaauugugaauucugagacaaauugcauagucacaaaagugaucguaaucucaggcaucaccac
......................................................................uUcauagucacaaaagugauc....................    2    1    seq
.......................................................................gcauagucacaaaagugauc....................    10    0    seq
```
